# Supplementary material for: Tailoring protein nanomechanics with chemical reactivity
Source: Nat Commun. 2017 Jun 6;8:15658. doi: 10.1038/ncomms15658 (PMC5467162; doi:10.1038/ncomms15658)
Supplement: Supplementary Information — Supplementary figures and supplementary tables. [file ncomms15658-s1.pdf]

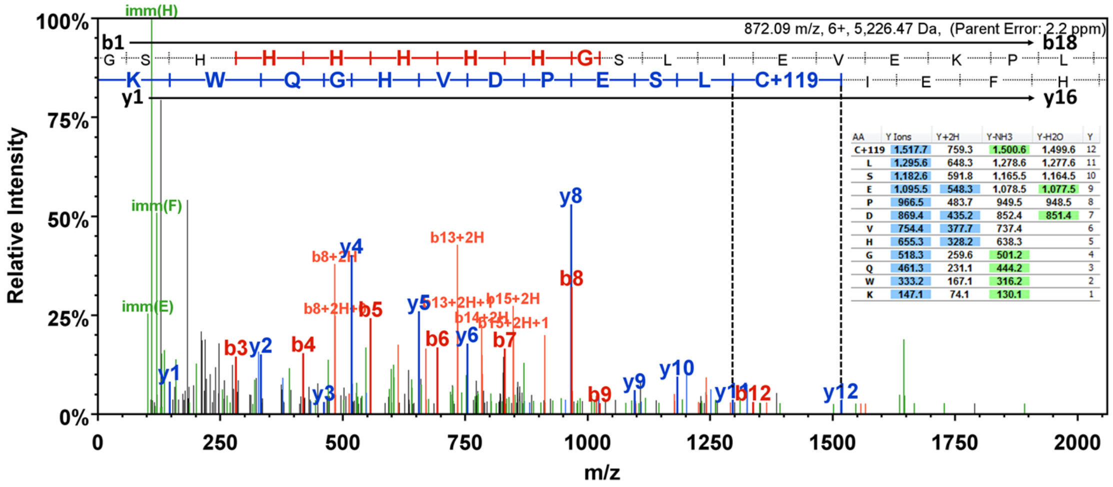

AA	Y Ions	Y+2H	Y-NH3	Y-H2O	Y
C+119	1,517.7	759.3	1,500.6	1,499.6	12
L	1,295.6	648.3	1,278.6	1,277.6	11
S	1,182.6	591.8	1,165.5	1,164.5	10
E	1,095.5	548.3	1,078.5	1,077.5	9
P	966.5	483.7	949.5	948.5	8
D	869.4	435.2	852.4	851.4	7
V	754.4	377.7	737.4		6
H	655.3	328.2	638.3		5
G	518.3	259.6	501.2		4
Q	461.3	231.1	444.2		3
W	333.2	167.1	316.2		2
K	147.1	74.1	130.1		1

Supplementary Figure 1. Evidence for a L-cysteine modification on a peptide with *m/z* 872.09^{6+}. HCD fragmentation followed by database searching has assigned a strong sequence string of consecutive ions from b4-b9 and y1-y12, which accurately confirms the peptide identification. A modification of 119 Da corresponding to an additional L-cysteine was assigned to the y12-ion (Cys24).

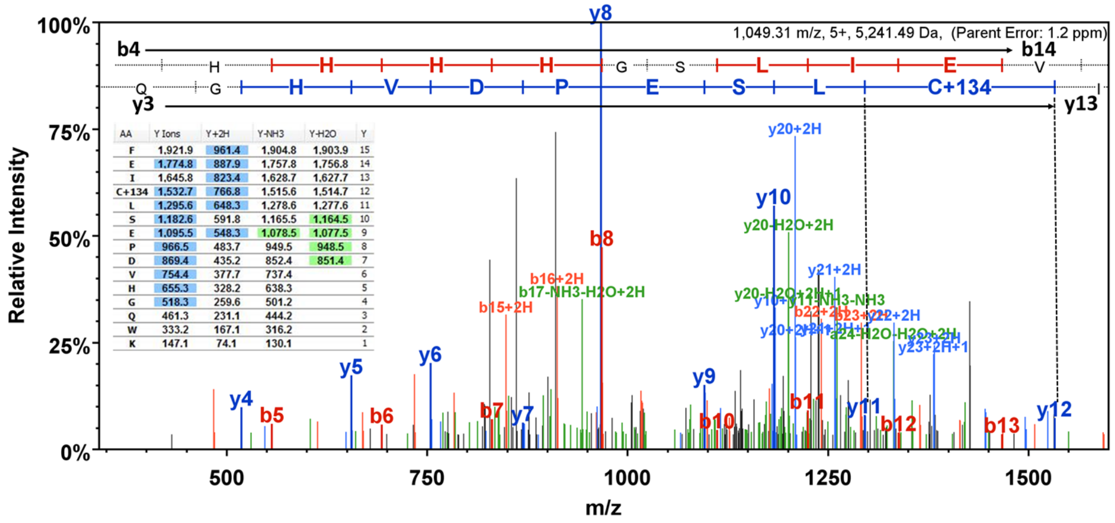

Supplementary Figure 2. Evidence for a S-homocysteine modification on a peptide with *m/z* 1049.31^{5+}. Evidence of a homocysteine modification on the cysteine-24 residue. A sequence tag of y-ions from y5-y12 and b-ions from b6-b8 and b11-b13 correctly identifies the peptide sequence of the protein at the N-terminus. An addition of 134 Da was detected on the Cys24 residue in the fragmentation spectra, which confirms homocysteine modification.

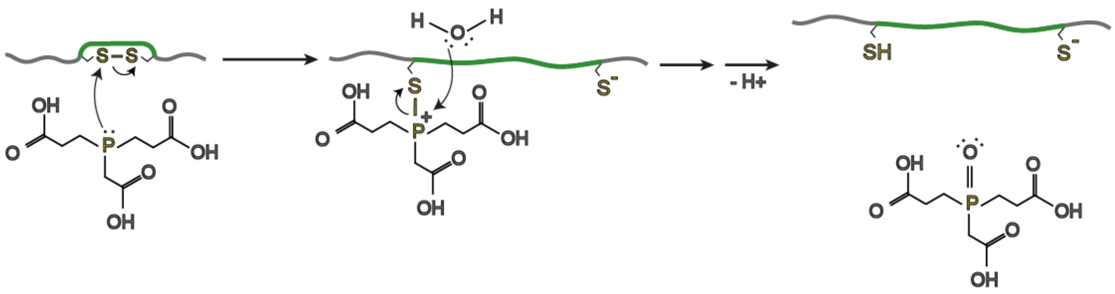

Supplementary Figure 3. Scheme of the protein disulfide reactivity with TCEP. TCEP induces disulfide bond reduction through an S_N2 chemical reaction to create an unstable S-P thioalkoxyphosphonium cation that can be rapidly attacked by water molecules. The chemical attack results in a fully extended protein that harbours two reduced cysteines which, upon force withdrawal, can successfully refold into a reduced protein conformation devoid of the mechanically stiff disulfide bond.

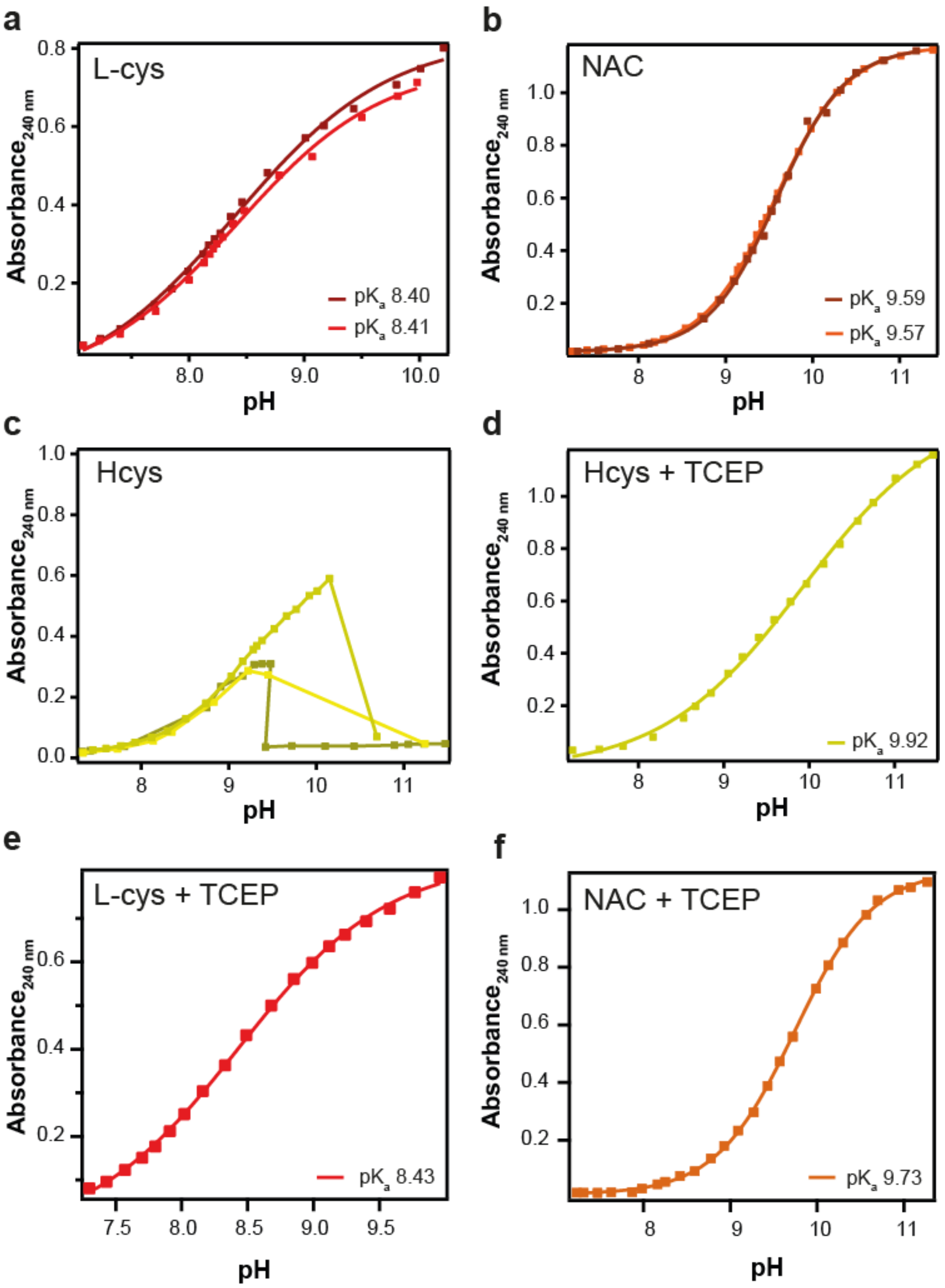

Supplementary Figure 4. pK_a determination for the different low molecular weight thiols. The determination of the p*K*a value for the different small thiols through absorbance measurements at λ= 240 nm proved highly reproducible within individual absorbance measurements as a function of pH. (**a**) A pK_a = 8.40 was measured for cysteine, (**b**) and a pK_a = 9.6 was obtained for NAC. (**c**) Homocysteine measurements showed a dramatic decrease in absorbance over time, especially a high pH values. Addition of 1 mM TCEP (highlighting the possible oxidation and homocysteine dimerization and precipitation) reversed the situation, allowing to measure a pK_a = 9.9. By contrast, adding 1 mM TCEP to the measuring solution did not affect the p*K*a determination of (**e**) cysteine or (**f**) NAC, consistent with their monomeric state in solution.

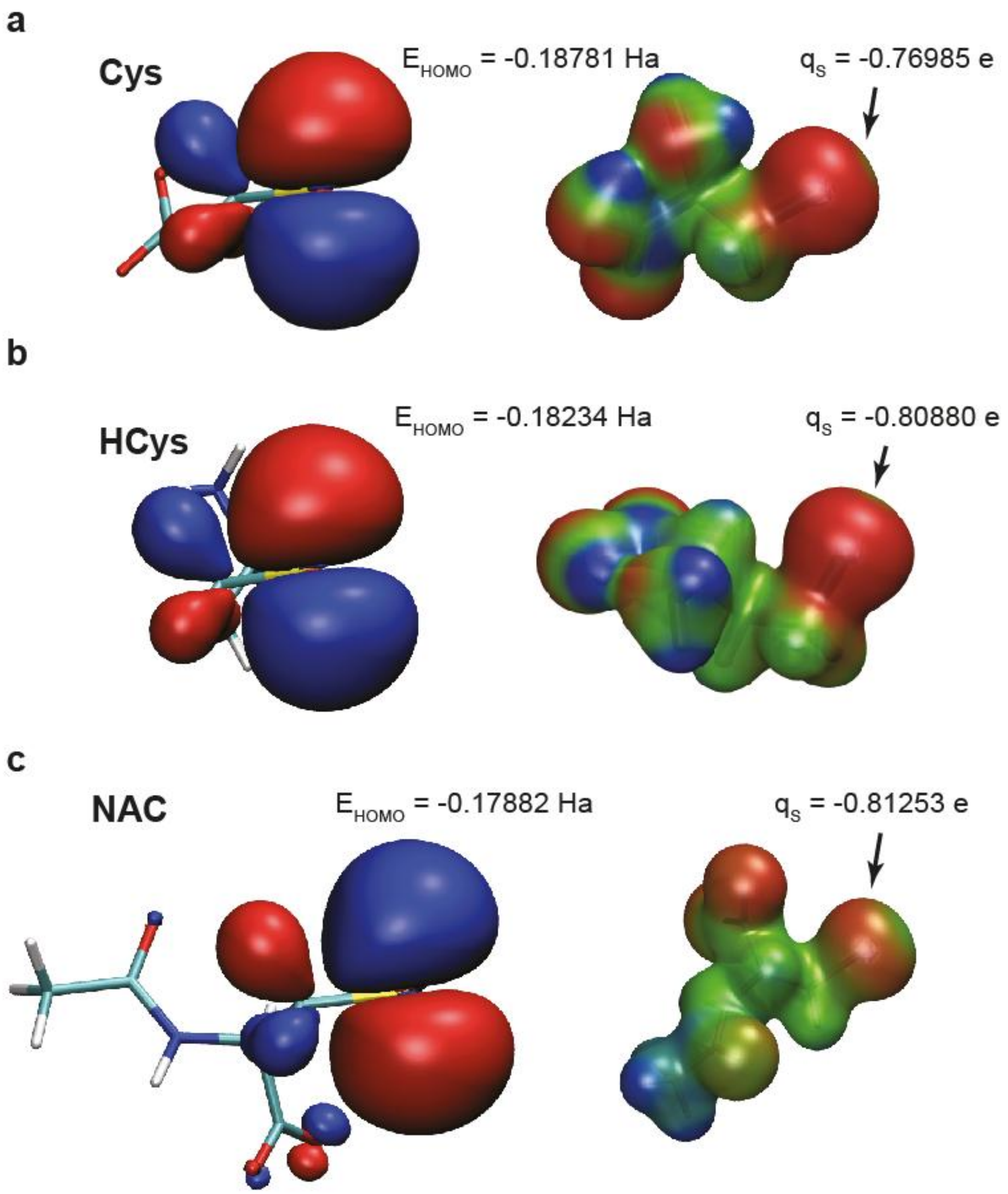

Supplementary Figure 5. (**a**) HOMO calculations (left) and electrostatic potentials (from red: negative, to blue: positive) mapped on an electronic isodensity (0.02) surface (right), together with the charges the sulfur atom for (a) L-cysteine, **(b)** homocysteine and (**c**) NAC, highlighting the good nucleophilic power of homocysteine and NAC.

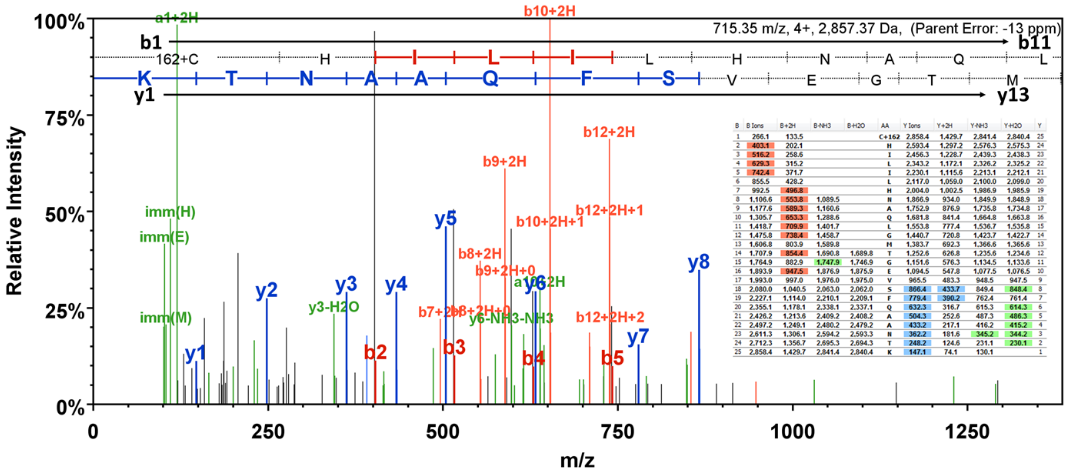

Supplementary Figure 6. Evidence for a NAC modification on a peptide with m/z 715.35^{4+}. Evidence for a CysNAC modification on the Cys55 residue. A sequence string of y-ions from y1-y8 and from b3-b5 confirms correct assignment of the peptide to the protein. The modification was assigned to the b1-ion and, although it was not covered in the spectra, the correct assignment of the b3-b5-ions after the modified residue is evidence for correct assignment to the cysteine residue at Cys55.

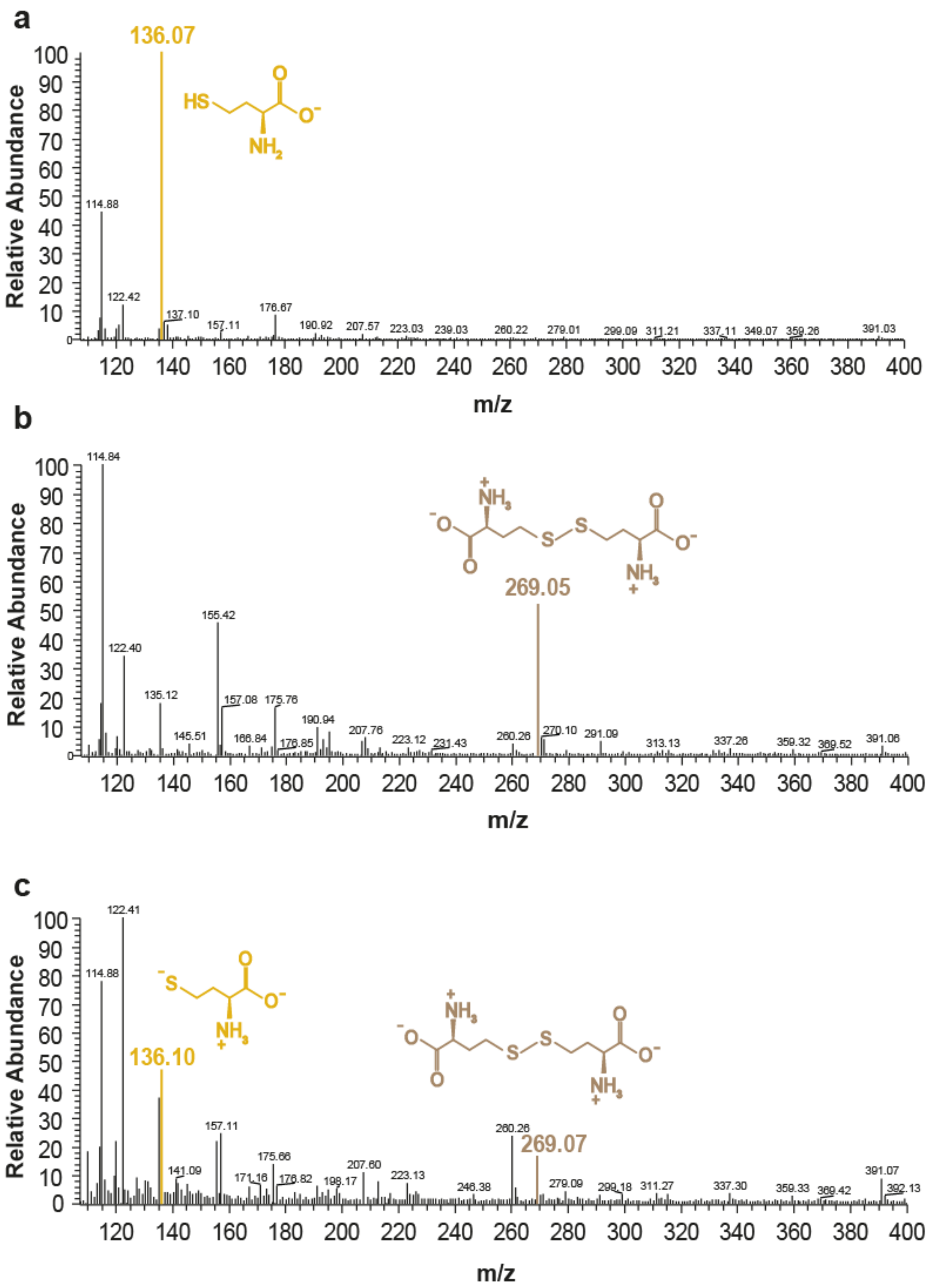

Supplementary Figure 7. Solution homocysteine undergoes fast oxygen-mediated dimerization, demonstrating the high tendency to form stable disulfide bonds. (**a**) A 62.5 µM homocysteine solution at pH = 7 does not dimerize under ambient conditions for 15 hours. (**b**) By contrast, exposing the same solution for 3 hours at pH = 9 induced full dimerization to homocystine (the homocysteine dimer). (**c**) Similar experiments in the presence of bubbling N_2 significantly delayed homocysteine appearance, thus suggesting a oxygen-induced homocysteine oxidation process. Crucially, homocystine is highly insoluble. These experiments highlight the high tendency of homocysteine to create disulfide bonds.

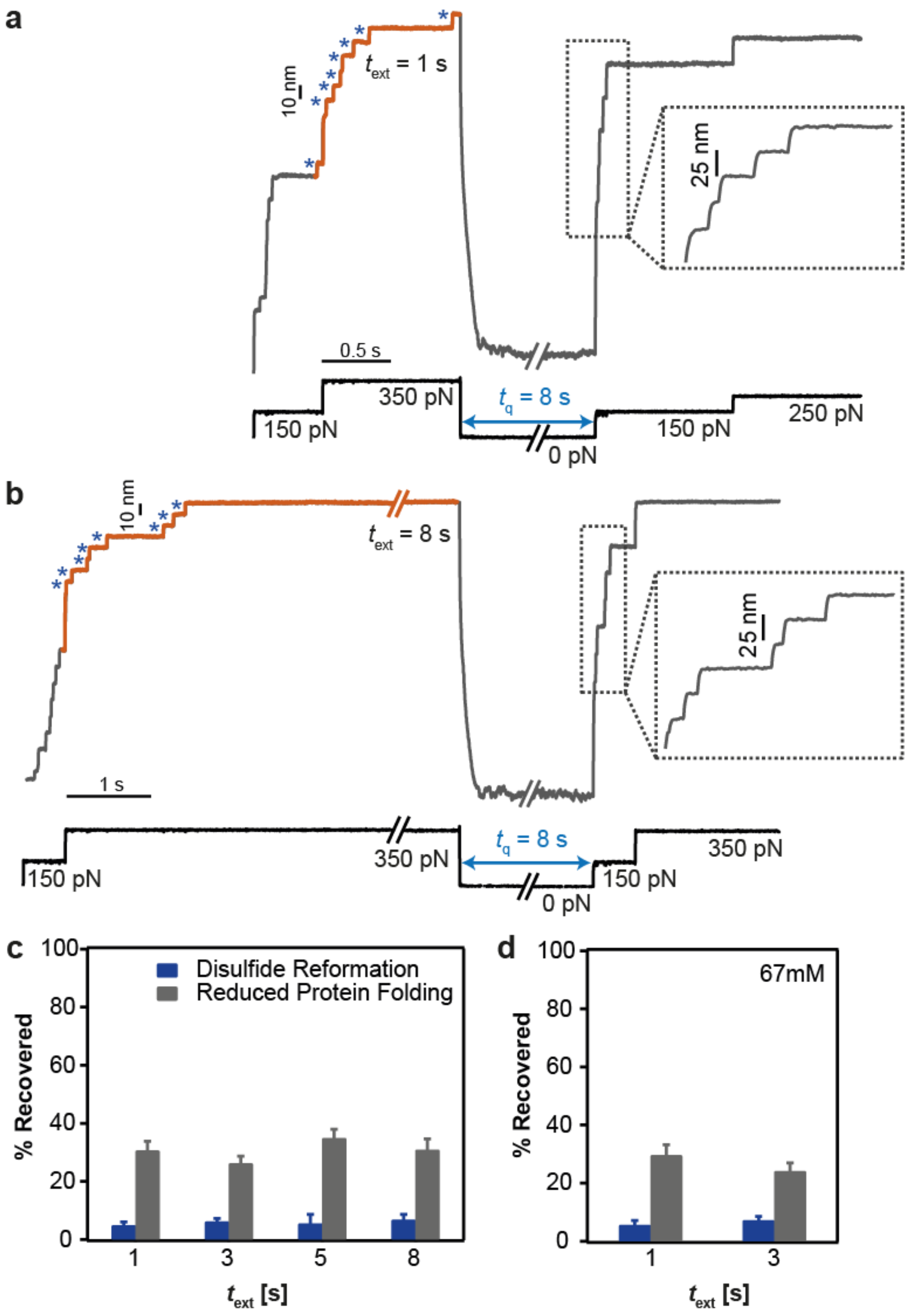

Supplementary Figure 8. NAC-mediated evolution of oxidative and reduced folding over time. The time-evolution presence of 10 nm steps (signature of oxidative folding) and 25 nm steps (hallmarking successful refolding of the reduced protein) in the test pulse was tested by changing the time, t_{ext}, the reduced protein is left unfolded and stretched, allowing it to react with solution nucleophiles. Surprisingly, leaving the protein extended for a short (**a**) t_{ext} = 1s and (**b**) long t_{ext} = 8s does not significantly change the folding percentages (**c**). Similarly, changing the solution concentration of NAC (± s.d) (**d**) does not have a significant effect on the folding fate of the protein. Hence, these experiments suggest that the reactivity of the unfolded and stretched protein occurs under thermodynamic —and not kinetic— control (± s.d).

Supplementary Table 1. Identification of peptide modifications by L-cysteine, Homocysteine and N-acetyl-cysteine. Mass spectrometry analysis by database searching and manual verification.

Sequence	m/z	Mr	Charge State	Intensity	Residue	Modification
^{2}GSHHHHHHGSLIEVEKPLYGVEVFVGETAHFEI**c**LSEPDVHGQWK^{46}	872.09	5,226.47	6+	455100	Cys24	L-Cys
^{66}**c**HILILHNAQLGMTGEVSFQAANTK^{90}	939.13	2,814.36	3+	29730	Cys55	L-Cys
^{2}GSHHHHHHGSLIEVEKPLYGVEVFVGETAHFEI**c**LSEPDVHGQWK^{46}	1049.31	5,241.49	5+	306700	Cys24	Hcys
^{66}**c**HILILHNAQLGMTGEVSFQAANTK^{90}	944.14	2,829.38	3+	144600	Cys55	Hcys
^{2}GSHHHHHHGSLIEVEKPLYGVEVFVGETAHFEI**c**LSEPDVHGQWK^{46}	1049.3	5,241.48	5+	1118000	Cys24	Hcys
^{66}**c**HILILHNAQLGMTGEVSFQAANTK^{90}	566.88	2,829.38	5+	242200	Cys55	Hcys
^{66}**c**HILILHNAQLGMTGEVSFQAANTK^{90}	1,415.70	2,829.39	2+	504100	Cys55	Hcys
^{47}LKGQPLAASPDAEIIEDGK**c**HILILHNAQLGMTGEVSFQAANTKSAANLK^{96}	896.79	5,374.70	6+	33960	Cys55	NAC
^{2}GSHHHHHHGSLIEVEKPLYGVEVFVGETAHFEI**c**LSEPDVHGQWK^{46}	879.26	5,269.50	6+	805900	Cys24	NAC
^{66}**c**HILILHNAQLGMTGEVSFQAANTK^{90}	715.35	2,857.37	4+	447700	Cys55	NAC
^{2}GSHHHHHHGSLIEVEKPLYGVEVFVGETAHFEI**c**LSEPDVHGQWK^{46}	874.59	5,241.47	6+	530100	Cys24	Hcys pH 7.6
^{66}**c**HILILHNAQLGMTGEVSFQAANTK^{90}	708.35	2,829.39	4+	293400	Cys55	Hcys pH 7.6

Supplementary Table 2. Sequences of all protein constructs, including six histidine residues at the beginning for purification. The polyprotein constructs for single molecule experiments have a cysteine tag at the end for attachment to the gold coverslides.

**Protein Construct**	**Polyprotein Sequence**
$(I27_{E24C\text{-}K55C})_8$	MRGSHHHHHHGSLIEVEKPLYGVEVFVGETAHFEICLSEPDVHGQW<br>KLKGQPLAASPDAEIIEDGKCHILILHNAQLGMTGEVSFQAANTKSA<br>ANLKVKELRSLIEVEKPLYGVEVFVGETAHFEICLSEPDVHGQWKLK<br>GQPLAASPDAEIIEDGKCHILILHNAQLGMTGEVSFQAANTKSAANL<br>KVKELRSLIEVEKPLYGVEVFVGETAHFEICLSEPDVHGQWKLKGQP<br>LAASPDAEIIEDGKCHILILHNAQLGMTGEVSFQAANTKSAANLKVK<br>ELRSLIEVEKPLYGVEVFVGETAHFEICLSEPDVHGQWKLKGQPLAA<br>SPDAEIIEDGKCHILILHNAQLGMTGEVSFQAANTKSAANLKVKELR<br>SLIEVEKPLYGVEVFVGETAHFEICLSEPDVHGQWKLKGQPLAASPD<br>AEIIEDGKCHILILHNAQLGMTGEVSFQAANTKSAANLKVKELRSLIE<br>VEKPLYGVEVFVGETAHFEICLSEPDVHGQWKLKGQPLAASPDAEII<br>EDGKCHILILHNAQLGMTGEVSFQAANTKSAANLKVKELRSLIEVEK<br>PLYGVEVFVGETAHFEICLSEPDVHGQWKLKGQPLAASPDAEIIEDG<br>KCHILILHNAQLGMTGEVSFQAANTKSAANLKVKELRSLIEVEKPLY<br>GVEVFVGETAHFEICLSEPDVHGQWKLKGQPLAASPDAEIIEDGKC<br>HILILHNAQLGMTGEVSFQAANTKSAANLKVKELRSRSCC
$(I27_{G32C\text{-}A75C})_8$	MRGSHHHHHHGSLIEVEKPLYGVEVFVGETAHFEIELSEPDVHCQW<br>KLKGQPLAASPDAEIIEDGKKHILILHNAQLGMTGEVSFQCANTKSA<br>ANLKVKELRSLIEVEKPLYGVEVFVGETAHFEIELSEPDVHCQWKLK<br>GQPLAASPDAEIIEDGKKHILILHNAQLGMTGEVSFQCANTKSAANL<br>KVKELRSLIEVEKPLYGVEVFVGETAHFEIELSEPDVHCQWKLKGQP<br>LAASPDAEIIEDGKKHILILHNAQLGMTGEVSFQCANTKSAANLKVK<br>ELRSLIEVEKPLYGVEVFVGETAHFEIELSEPDVHCQWKLKGQPLAA<br>SPDAEIIEDGKKHILILHNAQLGMTGEVSFQCANTKSAANLKVKELR<br>SLIEVEKPLYGVEVFVGETAHFEIELSEPDVHCQWKLKGQPLAASPD<br>AEIIEDGKKHILILHNAQLGMTGEVSFQCANTKSAANLKVKELRSLIE<br>VEKPLYGVEVFVGETAHFEIELSEPDVHCQWKLKGQPLAASPDAEII<br>EDGKKHILILHNAQLGMTGEVSFQCANTKSAANLKVKELRSLIEVEK<br>PLYGVEVFVGETAHFEIELSEPDVHCQWKLKGQPLAASPDAEIIEDG<br>KKHILILHNAQLGMTGEVSFQCANTKSAANLKVKELRSLIEVEKPLY<br>GVEVFVGETAHFEIELSEPDVHCQWKLKGQPLAASPDAEIIEDGKK<br>HILILHNAQLGMTGEVSFQCANTKSAANLKVKELRSCC
$I27_{E24C\text{-}K55C}$	MRGSHHHHHHGSLIEVEKPLYGVEVFVGETAHFEICLSEPDVHGQW<br>KLKGQPLAASPDAEIIEDGKCHILILHNAQLGMTGEVSFQAANTKSA<br>ANLKVKEL
